# Can ultrasound be the diagnostic modality to diagnosis anti-N-methyl-D-aspartate receptor encephalitis?

**DOI:** 10.1259/bjrcr.20220024

**Published:** 2022-09-12

**Authors:** Andrew Alaya, Frédérique van Dunné

**Affiliations:** 1 Gynaecology and Obstetrics Department, Haaglanden Medisch Centrum, The Hague, The Netherlands

## Abstract

Anti-N-methyl-D-aspartate receptor encephalitis is an auto-immune paraneoplastic disease of the limbic system. This syndrome may present itself as a range of psychiatric, neurological and autonomic features. It is associated with long-term morbidity and mortality. The syndrome is associated with ovarian teratoma affecting mainly young females. If the syndrome is identified at an early stage, then a good prognosis is expected. In this case report, the ultrasound performed by an experienced and competent operator was more sensitive than MRI in diagnosing a small ovarian teratoma. Following the removal of the teratoma and with appropriate immunotherapy the patient’s condition improved. In the clinical suspicion of anti-A-methyl-D-aspartate receptor encephalitis, the first modality of choice should be an ultrasound of the ovaria performed by an experienced and competent operator.

## Introduction

About 80% of patients with anti-N-methyl-D-aspartate receptor (anti-NMDAR) encephalitis are female. The incidence is dependent on age and ethnicity, with the highest occurrence between 18 and 36 years of age, and more prevalent among black than Caucasian females. This is in accordance with the prevalence of ovarian teratoma as the underlying tumour.^1^ Anti-NMDAR is a multistage illness that can start with physical symptoms such as headaches, fever, nausea and vomiting and can rapidly progress to severe psychiatric symptoms such as psychosis, memory loss, seizures and language disintegration. The illness can alternate between periods of agitation and catatonia, which can express itself in abnormal movements and autonomic instability leading to a state of unresponsiveness potentially requiring ventilatory support.^2^ MRI and ultrasound are the modalities of choice for detecting ovarian teratoma. This case report shows how ultrasound can be superior to MRI in the diagnosis of anti-NMDAR encephalitis.

## Case report

**Figure 1.**
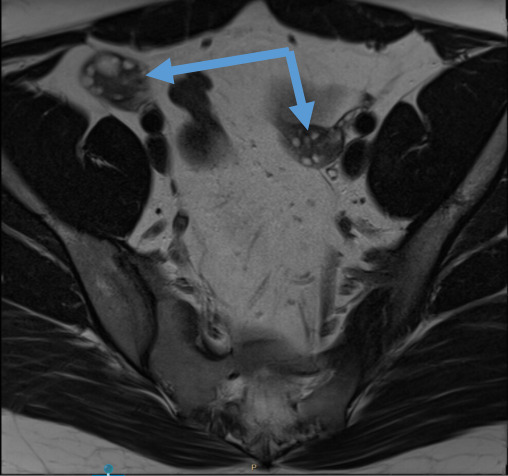
Pelvic MRI revealing normal ovaries (arrow).

A 27-year-old obese (BMI 41) female was admitted to the emergency department (ED) in a very aggressive and confused state, requiring restraint. The patient had an acute onset of mental confusion which started 4 days prior. She was restless, shouting, hallucinating with hypersexual behaviour and complaining of headaches. Neurological examination showed psychomotor instability with incoherent speech but no motor dysarthria. Her vital signs were normal. Basic blood evaluation was without abnormalities. Drug testing (blood, urine) was negative. A CT scan of the brain demonstrated no abnormality. Based on the clinical presentation and results so far, the differential diagnosis was viral encephalitis, intoxication, first symptoms of psychiatric abnormality or the possibility of anti-NMDAR encephalitis. The patient was transferred to the medical psychiatric unit where a lumber puncture was performed. Anti-viral therapy was commenced. However, this therapy was terminated 3 days later as the patient developed acute renal failure, most likely due to the administration of acyclovir. In the following 4 days, liquor test results came back positive for anti-NMDAR antibodies. MRI scan of the brain and pelvis (Figure 1) were performed under general anaesthesia. The result of both the brain and pelvis MRI were unremarkable, specifically normal ovaries were seen with respect to size and aspect and no teratoma was evident. A transabdominal ultrasound was performed revealing no abnormalities of the ovaries. A transvaginal scan was not preformed due to the non-consent of next of kin (parents).

On Day 7, the patient developed high temperature (39°C) and tachycardia (150 bpm) and was transferred to ICU to stabilize her condition. An electroencephalogram (EEG) revealed diffuse encephalitis. Due to the high suspicion of anti-NMDAR encephalitis with an ovarian source, a transvaginal ultrasound was again debated, however, the radiologist and gynaecologist thought a transvaginal ultrasound not necessary since the earlier pelvic MRI had revealed normal ovaries. They also argued their case considering the patient was a virgin and the ethical issue around such a decision.

**Figure 2.**
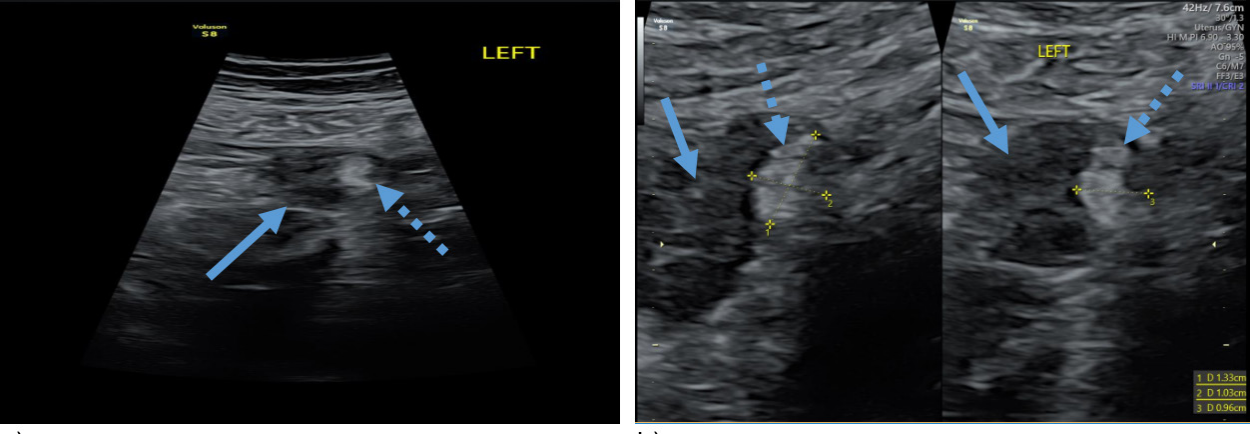
Transabdominal ultrasound revealing left ovary with a teratoma, (a) left ovary (arrow) with teratoma (dotted arrow), (b) measurement of a teratoma (dotted arrow) in the left ovary (arrow).

As her symptoms were not improving, a transvaginal ultrasound was again reconsidered on Day 18 in search for a cause of the antibodies. Permission was now obtained of the patient’s next of kin to perform both a transabdominal and a transvaginal ultrasound. The scan was performed by an experienced ultrasound operator and gynaecologist present with the patient under general anaesthesia in the operating theatre. The transabdominal ultrasound, that was performed first, showed a teratoma on the left ovary which was confirmed by transvaginal ultrasound. The teratoma was small, measuring 1.2 × 1 × 1 cm (Figure 2) and despite her high BMI could be visualised clearly.

**Figure 3.**
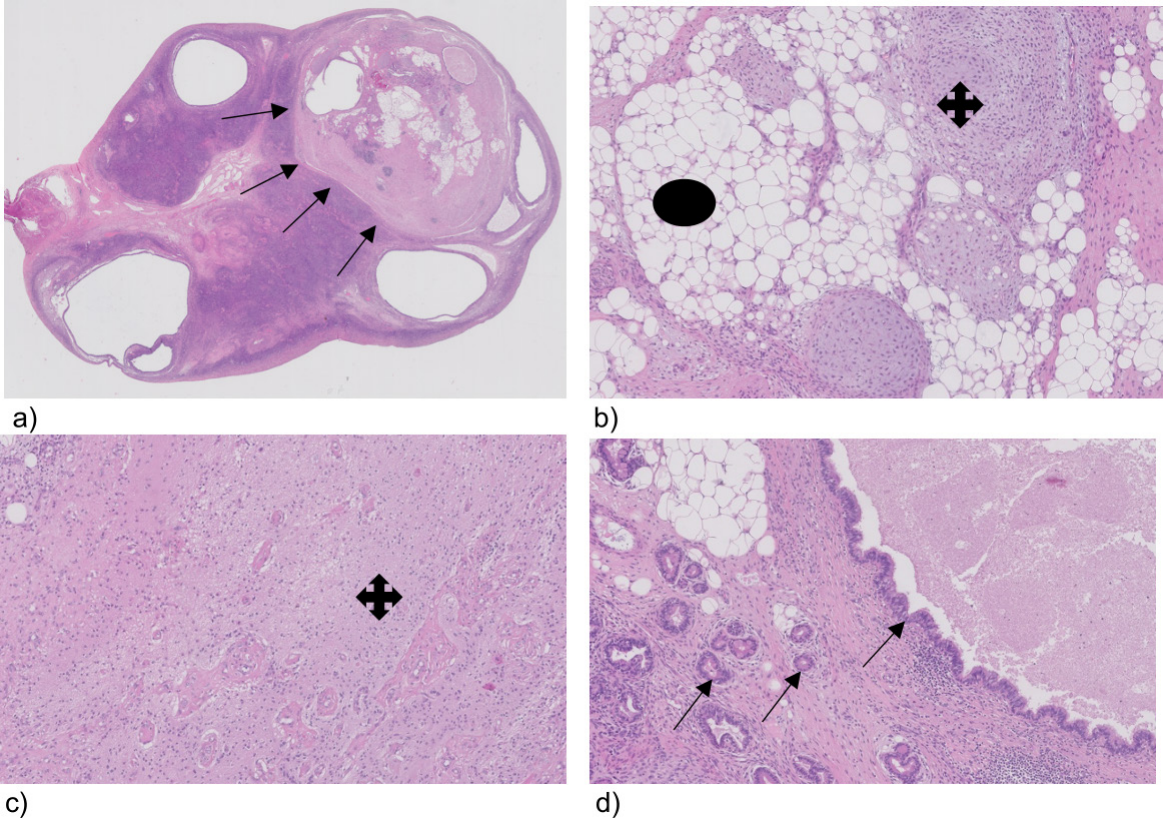
Tissue section from the lift ovary with a teratoma, (a) ovary with a teratoma (arrows), (b) teratoma, magnification 100, showing fat (circle) and cartilage (cross), (c) glial cell (cross) magnification 100, (d) glandular duct cells (arrows) magnification 100.

The following day, the patient underwent laparoscopic removal of the left ovary. Macroscopically the ovary was unremarkable. Pathohistological results, however, confirmed a mature teratoma with no immature characteristics (Figure 3).

In the 3 weeks following, the patient showed significant improvement of her symptoms. She was discharged after 6 weeks. At her check-up another 6 weeks later, her improvement was remarkable. She was living independently with little residual symptoms. Her speech had not completely recovered and she still has some long-term memory loss.

## Discussion

The number of reports indicating an association of anti-NMDAR encephalitis to ovarian teratoma has increased in developing countries since its identification and publication by Dalmau et al.^2^ Ovarian cystic teratomas are common benign tumours found in young asymptomatic females. Most do not cause complications. However, there is a 16% chance of ovarian torsion, a 1–5% chance of rupture or a 1–2% chance of malignant transformation.^3^ A very rare condition associated with teratomas is anti-NMDAR encephalitis. Anti-NMDAR encephalitis is caused by the presence of nervous tissue in the teratoma producing autoantibodies against NMDAR.^2^ As anti-NMDAR encephalitis is uncommon, diagnosis is often delayed, as neuropsychotic and infective encephalitis are more likely.^2^ Because prodromal symptoms are unspecific, the diagnosis takes time to manifest. Symptoms like headache, fever, gastrointestinal disorders and upper respiratory-tract symptoms progress to confusion, memory deficit, speech disorder, personality change, mood disturbances and psychosis. Therefore anti-NMDAR encephalitis will not be high in the differential diagnosis. As symptoms worsen and progress to facial and limb movement disorders, loss of consciousness, and seizures,^2^ a correct diagnosis and treatment is important. Due to the dominance of psychiatric symptoms, the majority of patients consults a psychiatrist first and can be misdiagnosed as newly onset psychiatric disorder. In this case, patients are treated with anti-psychotic drugs. Anti-NMDAR encephalitis patients do not respond to these drugs and can rapidly progress to late-stage symptoms with decreased responsiveness, tachycardia and urinary incontinence as observed in the patient in this case report.

Patients with these symptoms should be considered for diagnosis of encephalitis. MRI and EEG should be performed to obtain a specific diagnosis. The MRI may show normal brain in 67% of patients, whilst 90% of these patients have an abnormal EEG. The diagnosis of anti-NMDAR encephalitis is possible after the detection of anti-NMDAR antibodies in cerebrospinal fluid (CSF).^2^ This was also observed in the patient in this case report. If anti-NMDAR antibodies are detected in CSF, this finding should prompt imaging like MRI or ultrasound of the pelvis to evaluate the presence of an underlying teratoma, which could be responsible for the production of autoantibodies. An ovarian teratoma is found in 31–50% of females affected by anti-NMDAR encephalitis.^2^ Removal of the teratoma should be considered without delay.^4^ Most patients having a teratoma removed in time and are started on immunosuppressive treatment are found to have a significant improvement in their neurologic status.^2^

In this case report, the MRI and initial transabdominal ultrasound of the pelvis were reported as normal. Due to non-improvement of the symptoms, the neurologist persisted on performing a transvaginal ultrasound. The sensitivity of an ultrasound is dependent on many factors including the operator’s skill and patient anatomy. In this case, an experienced gynaecological ultrasound operator was asked to perform the second ultrasound examination. On transabdominal ultrasound, a teratoma of 1.2 × 1×1 cm was seen and confirmed by transvaginal ultrasound. The left ovary was removed laparoscopically. It is worthwhile noting the ultrasound image corresponded perfectly in size and location to the histological mass of the left ovary (Figures 2 and 3). After 3 weeks, the patient showed significant improvement. 6 weeks later, she was doing well, and recovery was almost complete.

## Conclusion

Ovarian teratoma can be associated with anti-NMDAR encephalitis in young females. Recognizing the disease as early as possible is crucial. Removal of the teratoma and treating the patient accordingly increases the possibility of a complete recovery. In this case report, an ultrasound was shown to be more sensitive than an MRI in detecting a small teratoma. However, the success of an ultrasound is dependent on the skills and experience of the operator.

## Learning points

- Anti-NMDAR encephalitis is a rare disease associated with ovarian teratoma.
- Recognizing the disease as early as possible may lead to complete recovery.
- Ultrasound is the modality of choice for determining ovarian teratoma.
- Ultrasound is operator-dependent. The skills and experience of the operator are of utmost importance.
